# Cutaneous Paracoccidioidomycosis

**DOI:** 10.4269/ajtmh.15-0062

**Published:** 2015-09-02

**Authors:** Juan Carlos Cataño, Milena Morales

**Affiliations:** Infectious Diseases Section, Internal Medicine Department, University of Antioquia School of Medicine, Medellín, Colombia; Infectious Diseases Section, Las Vegas Clinic, Medellín, Colombia

**Figure 1.**
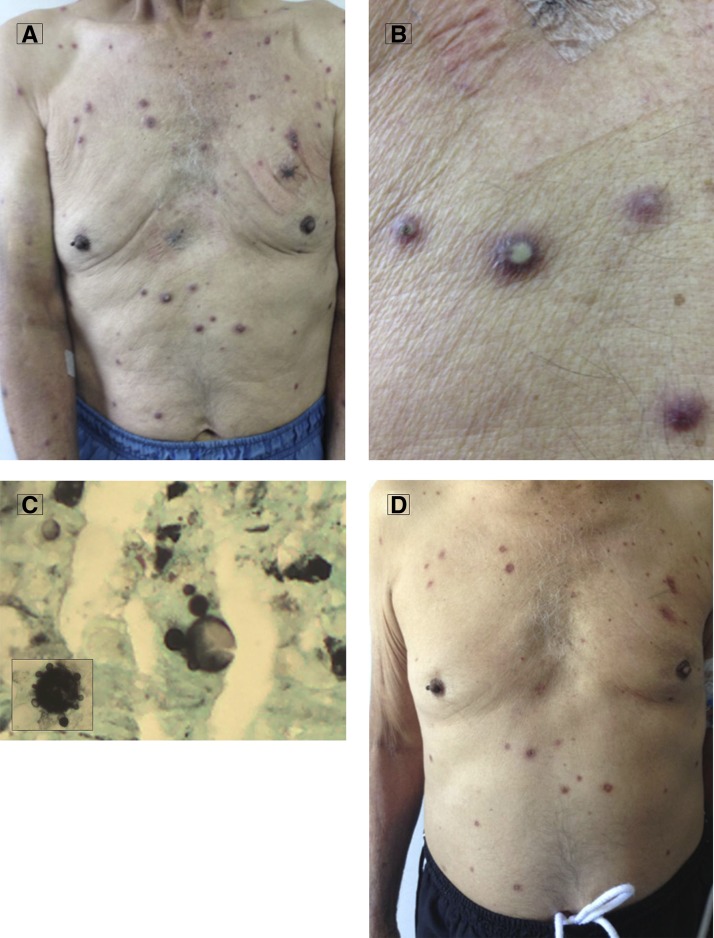
(**A**) Disseminated pustular skin lesions; (**B**) disseminated pustular skin lesions (close); (**C**) multiple, narrow base, budding yeast cells “steering wheels” of *Paracoccidioides brasiliensis* on Grocott's methenamine silver (GMS) stain; (**D**) after treatment clinical improvement.

A 69-year-old man, with no remarkable medical history, had been working his entire life as a farmer in a rural area of Colombia. He presented with 3 months of fever, multiple cutaneous lesions, and 10 kg weight loss. Physical examination demonstrated that he was malnourished, pale, and had disseminated pustular skin lesions on the trunk and arms (Figure 1A and B). The remainder of the exam did not show oral, chest, or abdominal findings. Computed chest and abdomen tomography did not reveal lung or intra-abdominal lesions. Human immunodeficiency virus (HIV) serology was positive, CD4 T-cell count was 23 cells/mL, and HIV viral load was 470.000 copies/mL. Microscopic examination of skin biopsies demonstrated multiple, narrow base, budding yeast cells—the “steering wheels” of *Paracoccidioides brasiliensis* on Grocott's methenamine silver (GMS) staining (Figure 1C). Culture on Saboreaud's medium confirmed the mycological diagnosis. Staining and culture for acid-fast bacilli were negative. Treatment with amphotericin B deoxycholate for 20 days (total 1 g) led to significant clinical improvement (Figure 1D). Follow-up treatment included oral trimethoprim/sulfamethoxazole and antiretroviral medication, without any relapse during follow-up. Paracoccidioidomycosis (or South American blastomycosis) is a systemic mycosis of high prevalence in Latin America, caused by dimorphic fungus *Paracoccidioides brasiliensis*. It has different clinical forms, and may affect any organ or system, but the cutaneous involvement seen in this case is most common in the chronic form of the disease. Paracoccidioidomycosis is uncommon in HIV-infected patients, perhaps partly because of the use of trimethoprim/sulfamethoxazole as prophylaxis for *Pneumocystis* infection, which this patient did not receive, but with the HIV pandemic, a larger number of paracoccidioidomycosis–HIV coinfection cases have to be expected, since currently HIV transmission has taken on a rural character, including, in South America, regions where the *Paracoccidioides brasiliensis* is found.^1^–^4^
